# Physicochemical Characterization and Asymmetric Catalytic Properties of New Biobased Organocatalytic Surfactants

**DOI:** 10.3390/molecules30020216

**Published:** 2025-01-07

**Authors:** Elliot Calbrix, Pascale de Caro, Sophie Thiebaud-Roux, Christine Cecutti, Emeline Vedrenne

**Affiliations:** Laboratoire de Chimie Agro-Industrielle (LCA), Université de Toulouse, INRAE, 4 allée Emile Monso, 31030 Toulouse, France; elliot.calbrix@ensiacet.fr (E.C.); pascale.decaro@ensiacet.fr (P.d.C.); sophie.thiebaudroux@ensiacet.fr (S.T.-R.); christine.cecutti@ensiacet.fr (C.C.)

**Keywords:** surfactant, biobased, green chemistry, organocatalyst

## Abstract

In organic synthesis, the solvent is the chemical compound that represents the largest proportion of the process. However, conventional solvents are often toxic and dangerous for the environment, and an interesting alternative is to replace them by water. In this context, catalyst surfactants allow both organic reagents in water to be solubilized and organic reactions to be catalyzed. This article describes the synthesis of new biobased organocatalytic surfactants soluble in water, composed of a hydrocarbon chain grafted onto an imidazolidinone moiety. The imidazolidinone moiety acts as catalyst, but also as the polar head of the surfactant, while the fatty chain constitutes the hydrophobic tail. The five steps of the synthesis were optimized, respecting the principles of green chemistry, and two organocatalytic surfactants were obtained with a good selectivity. Surface properties in an aqueous medium were then evaluated with the use of tensiometric analysis. Their molecular organization in vesicles was characterized by Dynamic Light Scattering. The presence of vesicles allows reactions to be carried out in an organized aqueous medium. Model catalytic reactions performed in aqueous medium validated the feasibility of replacing conventional hazardous organic solvents. The newly synthesized biobased surfactants showed satisfactory catalytic activity and allowed the expected products to be obtained with good enantioselectivity.

## 1. Introduction

One of the chemical industry’s most frequently cited problems is the production of large quantities of waste, due, in particular, to the use of organic solvents, most of which are not recycled. In the pharmaceutical industry, for example, solvents account for more than 80% by mass of the compounds involved [1]. In addition, most of the organic solvents commonly used in laboratories and industry are toxic to humans and/or the environment. A major challenge is, therefore, to find alternatives solvents, in line with the principles of green chemistry [2].

However, the insolubility of many organic compounds limits the application of water as a solvent. In particular, low yields or selectivities have been obtained when water is used as a reaction medium in catalyzed reactions. To overcome these drawbacks, strategies based on the use of surfactants can be envisaged. The organization of surfactants in water allows the solubilization of organic compounds in the surrounding aqueous phase [3], eliminating the need for organic solvents, as the reactions take place in the hydrophobic core of the formed aggregates (micelles, vesicles, etc.).

Moreover, organocatalysis is an extremely powerful tool that has been developing steadily since the early 2000s, due to the lower toxicity of organocatalysts compared to metal catalysts, their lower cost, and better compatibility with water [4].

In recent years, a great deal of research has been carried out into the design of molecules that can play both the role of a surfactant and an organocatalyst [5,6,7,8,9]. The advantage of these molecules is that they reduce the amount of chemical compounds involved (no excess reagent), while allowing organocatalyzed reactions to take place in water.

The vast majority of organocatalytic surfactants are proline or threonine derivatives. They have the advantage that they can be used directly in water. While these surface-active organocatalysts allowed enantio-enriched molecules to be obtained, demonstrating the effectiveness of the concept, the results obtained in terms of yield and enantiomeric excess seem to be substrate-dependent. However, the development of new efficient asymmetric synthesis methods is crucial, since a very large number of molecules, whether biologically active compounds for the pharmaceutical and agrochemical industries, food additives, or flavoring agents, require pure enantiomers [10]. This opens the way for the development of new organocatalytic surfactants (other than proline or threonine derivatives) that would allow good yields and enantiomeric excesses for a wide range of reactions.

Imidazolidinones represent a specific type of organocatalyst developed by D. MacMillan. In recognition of their pioneering work in this field, he and B. List were awarded the Nobel Prize in Chemistry in 2021. The imidazolidinone moiety behaves like a Lewis acid via the formation of an iminium [11] and can be obtained from a phenylalanine derivative (amino acid). The geometry of imidazolidinones also allows asymmetric catalysis to be performed on a wide range of chemical reactions.

**Figure 1:**
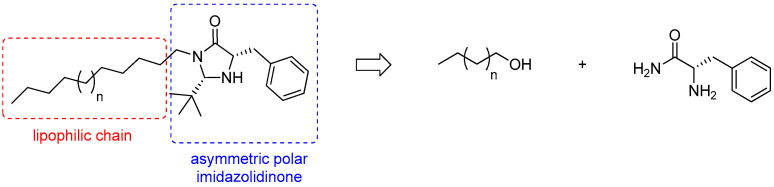
Retrosynthetic pathway of the targeted surfactant.

In this context, the aim of this work is to synthesize chiral organocatalysts with surfactant properties for asymmetric reactions in water, thus, meeting green chemistry criteria. We, thus, propose herein the synthesis of a new asymmetric organocatalytic surfactant, consisting of the imidazolidinone organocatalyst moiety and a fatty chain (Figure 1). This lipophilic moiety is readily available in the lipids of brown algae and was chosen as a follow-up to previous work on the valorization of these algae [12].

Syntheses work carried out according to the principles of green chemistry led to the production of two molecules whose surface-active and catalytic properties for enantioselective reactions were evaluated.

## 2. Results and Discussion

### 2.1. Synthesis

**Figure 2:**
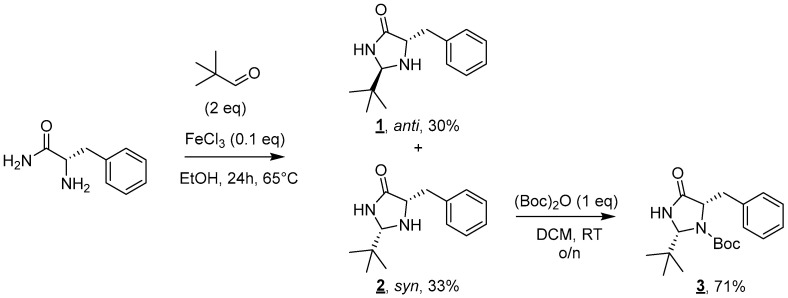
Synthesis of imidazolidinones **1**, **2,** and **3**.

The proposed synthesis relies on the grafting of a lipophilic chain on a polar catalytic moiety, here a 4-imidazolidinone [13,14]. The first step of this convergent synthesis involves the formation of the imidazolidinone moiety (Figure 2). The reaction of *L*-phenylalaninamide with two equivalents of pivaldehyde in the presence of a catalytic amount of iron(III) chloride led to the formation of a 1:1 *syn*/*anti* mixture of diastereoisomers **1** and **2** after 24 h at 65 °C [15]. Diastereoisomers **1** and **2** were efficiently separated on a silica gel column and, respectively, obtained in 30% and 33% yields.

As only the syn diastereoisomer was described in the literature to present an interesting catalytic activity [11], molecule **1** was discarded for the rest of the synthesis. The amine moiety of imidazolidinone **2** was then Boc-protected to deactivate this group prior to any further functionalization. This protection was performed using one equivalent of Boc_2_O in DCM at room temperature overnight. Molecule **3** was obtained after purification in 71% yield [16].

In order to graft a lipophilic chain on the obtained imidazolidione **3**, two fatty alcohols, decan-1-ol and hexadecan-1-ol, first needed to be converted into reactive mesylated alcohols (Table 1). The corresponding reactions were run in the presence of a 1.5 equivalent of MsCl and a 2 equivalent of a base in DMC for 7 h [17]. After an acidic treatment, mesylated alcohols **4** and **5** were, respectively, obtained in 95% and 70% yields without further purification.

**Figure 3:**
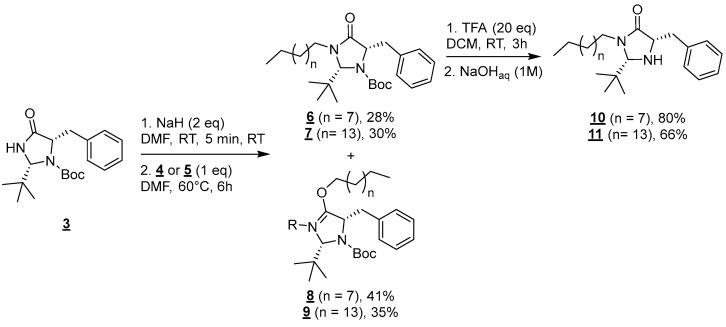
Formation of compounds **6** to **11**.

Molecules **4** and **5** were then engaged in the grafting step: the nucleophilic substitution between amide **3** and mesylated alcohols **4** or **5** was carried out in the presence of NaH (60% dispersed in mineral oil) in anhydrous DMF at 60 °C for 6 h (Figure 3) [18].

In both cases, this reaction led to the formation of a mixture of regioisomers resulting from competitive N (**6** and **7**) and O-alkylations (**8** and **9**) [19]. Those regioisomers were separable on silica gel columns and compounds **6** and **8** (n = 7) and **7**, and **9** (n = 13) were, respectively, obtained in 28, 41, 30, and 35% yields. In this paper, we chose to focus on the synthesis of surfactants derived from the N-alkylation products **6** and **7**. The functionalization of the O-alkylation derivatives **8** and **9** will be published in due time.

Finaly, the deprotection of amine groups of compounds **6** and **7** was carried out using trifluoroacetic acid (TFA) in DCM for 1 h at room temperature [20]. Targeted molecules **10** and **11** were, respectively, obtained after a base workup in 80 and 66% yields.

We then decided to evaluate the surfactant properties of the synthesized molecules.

### 2.2. Solubility in Water

**Figure 4:**
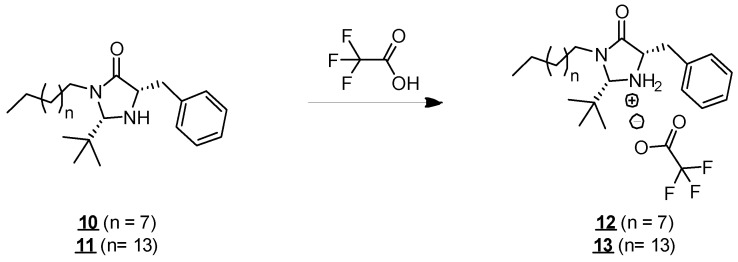
Formation of compounds **12** and **13**.

Molecules **10** and **11** have an atypical structure for surfactants. They have two main hydrophobic groups (C10 and C16 hydrocarbon chains and an aromatic group) and a hydrophilic center (imidazolidinone unit). They are, therefore, molecules with a more lipophilic character. Their solubility in organic solvents, such as cyclohexane, ethyl acetate, and dichloromethane was observed during the various reactions in which they were involved and the corresponding purification stages. However, the molecules remained insoluble in water from 25 to 45 °C, even after vigorous stirring, forming a fatty organic immiscible phase above the water. In order to make the molecules soluble in water, we protonated them by an acid treatment (Figure 4). The effect of protonation on the solubility of molecules in water has already been described in the literature, particularly for long-chain amines, such as hexadecanamide, which is poorly soluble in water [21] but could be solubilized at higher concentrations in its protonated form [22,23]. In our case, the addition of trifluoroacetic acid allowed us to convert the amine function into its ammonium form, thus, giving a positive charge to the imidazolidinone unit. The resulting molecules (**12** and **13**, respectively**)** presented solubility in water at 25 °C up to 3 g·L^−1^ and 1 g·L^−1^ for molecules **12** and **13**, respectively, allowing the study of their surface activity.

### 2.3. Surface Tension

**Figure 5:**
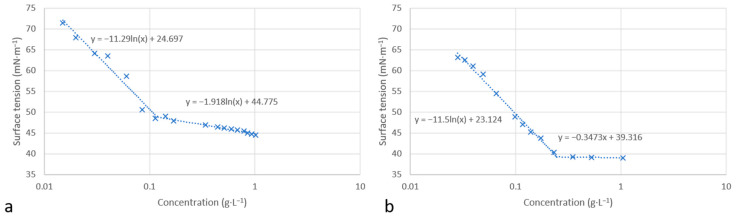
Surface tension versus concentration for compounds **12** (**a**) and **13** (**b**).

The surface tension in water was determined using the Wilhelmy plate method at 25 °C. Figure 5 presents the variation in the surface tension versus concentration for molecules **12** and **13**. Similar curves were obtained in both cases, showing the decrease in water/air surface tension with increasing surfactant concentration. An inflection point was observed for each molecule corresponding to a critical aggregation concentration, CAC_w_, in water. Above this concentration, the surface tension remains constant.

In order to investigate the adsorption of molecules at the water/air interface, we used the Gibbs equation to calculate the maximum saturated adsorption capacity Γ_max_ and the minimum molecular occupied area A_min_ (Equations (1) and (2)).

(1) $$\Gamma \max=\;-\frac{1}{nRT}{\left(\frac{\partial \gamma }{\partial \mathrm{lnC}}\right)}_{T}$$

(2) $$A_{\min}=\frac{10^{20}}{N_{a}\Gamma_{\max}}$$

where C is the surfactant concentration (M), γ is the surface tension (mN·m^−1^), and *n* takes the value of 2 for an ionic surfactant with a monovalent cation; *T* represents the temperature (K), and *R* is the ideal gas constant (8.314 J·mol^−1^·K^−1^). *N_a_* is the Avogadro constant (6.022 × 10^23^ mol^−1^).

As shown in Table 2, the CAC_w_ values for molecules **12** and **13** are quite close to those of conventional surfactants, in particular Tween 20 and Triton X100. The measured values of the surface tension, γ_w_, at the critical association concentration of both molecules also show a surface activity similar to the three conventional surfactants (Tween 20, Triton X 100, and CTAB).

It can be observed that molecule **13**, with a longer alkyl chain, has a higher occupied molecular area A_min_ compared to molecule **12**. A similar effect of the hydrocarbon chain length on the A_min_ value has already been described by Man [30]. The long chains are more flexible and can induce a larger occupied area at the water/air interface. Furthermore, the calculated values of A_min_ and Γ_max_ are in the same order of magnitude as those of the three conventional surfactants chosen as references (Tween 20, Triton X 100 and CTAB).

### 2.4. Aggregation Behavior in Water

**Figure 6:**
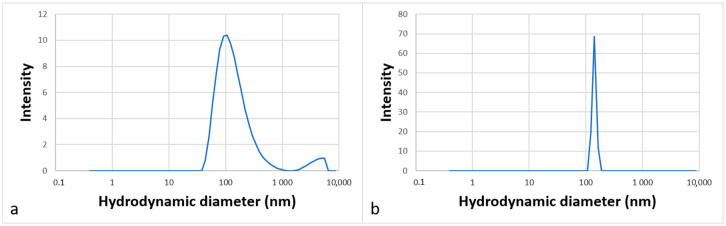
Partial size distribution of aggregates formed in an aqueous solution of surfactants **12 **(**a**) and **13** (**b**) at 3 g·L^−1^ and 1 g·L^−1^, respectively.

The aggregation behavior was studied by DLS analysis in water, which corresponds to the medium of the future catalytic reactions. Figure 6 shows the intensity-weighted size distribution (a) for molecule **12** at 3 g·L^−1^ and (b) for molecule **13** at 1 g·L^−1^. The curves show unimodal size distributions.

The diameters of the particles measured were around 120 ± 2 nm for molecule **12** and around 140 ± 21 nm for molecule **13**, indicating that molecules can self-assemble in water to form large aggregates that can be assimilated to vesicles. Such organized structures dispersed in water are suitable for carrying out organic reactions at their core [4].

### 2.5. Asymmetric Catalytic Activity of Compounds ***12*** and ***13*** in Water

**Figure 7:**
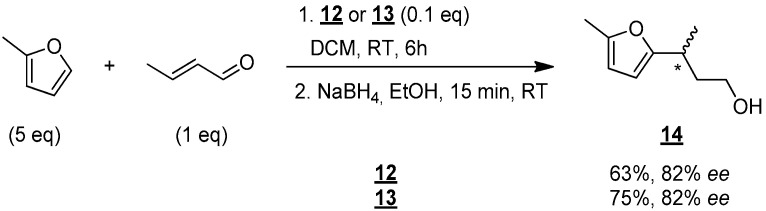
Enantioselective synthesis of compound **14 **in DCM. * indicates the asymmetric carbon.

The performance of catalysts **12** and **13** were tested in enantioselective Friedel–Crafts reaction (Figure 7) [15]: crotonaldehyde (1 equiv), 2-methoxythiophene (5 equiv) were stirred in the presence of a catalytic amount of **12** or **13** (0.1 equiv) in DCM at room temperature for 6 h. The resulting aldehyde was then reduced to give alcohol **14**, which was finally analyzed to determine the enantioselectivity of the reaction.

Alcohol **14** was then obtained in good yield of 63% and an encouraging enantiomeric excess of 82% with catalyst **12**. The use of catalyst **13** led to the isolation of **14** in 75% yield and an identical enantiomeric excess of 82%. These results, therefore, demonstrate that molecules **12** and **13** do indeed catalyze the Friedel–Crafts reaction, and they do so stereoselectively.

**Figure 8:**
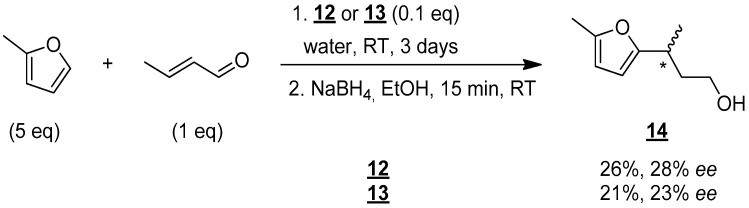
Enantioselective synthesis of compound **14** in water. * indicates the asymmetric carbon.

Given these promising results, the use of these new organocatalytic surfactants, **12** and **13**, was tested in water (Figure 8).

Under the same conditions described above (number of equivalents, temperature), vigorous stirring was required when water was used as the solvent in order to obtain a good emulsion. ^1^H NMR monitoring of the reaction also showed that a reaction time of 3 days was required to obtain reasonable yields. Alcohol **14** was then isolated in yields of 26% and 21% using molecules **12** and **13**, respectively. These results show that **12** and **13** really act as organocatalytic surfactants. However, a decrease in chiral activity was observed as enantiomeric excesses of 28% and 23% were obtained with **12** and **13**, respectively. Despite this reduction in enantiomeric excess, the results are still very encouraging, and a more detailed study is underway to modify the reaction conditions to obtain enantiomerically pure compounds. The results will be published in due time.

## 3. Materials and Methods

### 3.1. Experimental Apparatus

^1^H NMR spectra were acquired with a FOURIER300 (300 MHz) or an Avance III HD 500 (500 MHz) spectrometer from Brüker Coporation (Karlsruhe, Germany). The chemical shifts, δ, are expressed in parts per million (ppm) and referenced to tetramethylsilane at 0 ppm. Coupling constants, *J*, are expressed in Hertz (Hz). Data are reported as follows: δ, multiplicity (br: broad, s: singlet, d: doublet, dd: doublet of doublet, t: triplet, q, quadruplet, and m: multiplet), *J*, integration, and assignment. All samples were diluted in deuterochloroform.

^13^C NMR spectra were recorded on the same instrument at 75 or 126 MHz. Chemical shifts are referenced to the central peak of residual CDCl_3_ (77.2 ppm).

Infrared (IR) spectra were recorded on a Spectrum 65 FT-IT spectrometer from Perkin SPE 263 IR, PerkinElmer (Villepinte, France) in ATR mode. Wavenumbers are expressed in cm^−1^.

High Resolution Mass Spectra (HRMS) were obtained on a GCT Premier (Waters, Milford, MA, USA) mass spectrometer via direct introduction. Analyses were performed at the Institut Chimique de Toulouse, Université Paul Sabatier, Toulouse, France.

The tensiometer was an automatic 3S GBX scientific instrument (Loire, France). Particle size analyses were performed on a Zetasizer Nano-ZS, Malvern Panalytical Instrument, Ltd. (Westborough, MA, USA).

### 3.2. Synthesis Experimental Procedure

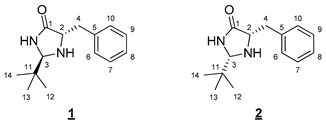

(2*R*,5*S*)-5-Benzyl-2-(*tert*-butyl)imidazolidin-4-one (**1**) and (2*S*,5*S*)-5-benzyl-2-(*tert*-butyl)imidazolidin-4-one (**2**)

To a solution of 0.998 g (6.08 mmol, 1 equiv) of L-phenylalaninamide in 30 mL of ethanol, the following were added: 0.105 g (0.61 mmol, 0.1 equiv) of FeCl_3_ and 1.45 mL (13.40 mmol, 2.2 equiv) of pivaldehyde. The resulting mixture was stirred at 65 °C for 24 h, and then the volatile compounds were evaporated in a rotary evaporator. Ethyl acetate was added to the medium, which was then filtered through Celite. The volatile compounds were then evaporated in a rotary evaporator. The crude product was finally purified by flash chromatography on silica gel (cyclohexane/EtOAc 5:5 (*v*/*v*)) to give 0.423 mg of anti **1** diastereoisomer (30%, white solid) and 0.471 mg of syn **2** diastereoisomer (33%, white solid).

***Anti* 1** diastereoisomer (Rf 0.40 (cyclohexane/EtOAc 5:5 (*v*/*v*)): ^1^H NMR (300 MHz, CDCl_3_) δ 7.40 (br s, 1H, NH), 7.35–7.21 (m, 5H, H_6–10_), 4.03 (d, *J* = 1.9 Hz, 1H, H_3_), 3.80 (ddd, *J* = 6.6, 4.4, 1.9 Hz, 1H, H_2_), 3.10 (dd, *J* = 14.1, 4.4 Hz, 1H, H_4_), 2.94 (dd, *J* = 14.1, 6.6 Hz, 1H, H_4_), 0.86 (s, 9H, H_12–14_). ^13^C NMR (75 MHz, CDCl_3_) δ 177.8 (C_1_), 137.2 (C_5_), 129.5 (C_6_, C _10_), 128.7 (C_7_, C_9_), 126.8 (C_8_), 77.8 (C_3_), 60.0 (C_2_), 38.0 (C_4_), 36.1 (C_11_), 24.2 (s, C_12–14_). IR (KBr): 3357, 3196, 2967, 2866, 1688, 1454, 1376, 756, 700 cm^−1^. HRMS (DCI-CH_4_) Calculated for C_14_H_21_N_2_O 233.1654 [M]^+^, found 233.1642.
***Syn* 2** diastereoisomer (Rf 0.15 (cyclohexane/EtOAc 5:5 (*v*/*v*)): ^1^H NMR (300 MHz, CDCl_3_) δ 7.81 (br s, 1H, NH), 7.36–7.17 (m, 5H, H_6–10_) 4.25 (d, *J* = 1.5 Hz, 1H, H_3_), 3.81 (ddd, *J* = 7.5, 4.0, 1.5 Hz, 1H, H_2_), 3.12 (dd, *J* = 13.7, 4.0 Hz, 1H, H_4_), 2.92 (dd, *J* = 13.7, 7.5 Hz, 1H, H_4_), 0.80 (s, 9H, H_12–14_). ^13^C NMR (75 MHz, CDCl_3_) δ 177.6 (C_1_), 137.9 (C_5_), 129.6 (C_6_, C_10_), 128.6 (C_7_, C_9_), 126.7 (C_8_), 77.3 (C_3_), 60.3 (C_2_), 37.7 (C_4_), 34.0 (C_11_), 24.3 (C_12–14_). IR (KBr): 3342, 3229, 2955, 2865, 1705, 1454, 1341, 730, 699 cm^−1^. HRMS (DCI-CH_4_): Calculated for C_14_H_21_N_2_O 233.1654 [M]^+^, found 233.1650.

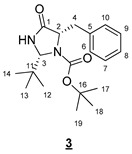

*tert*-Butyl (2*R*,5*S*)-5-benzyl-2-(*tert*-butyl)-4-oxoimidazolidine-1-carboxylate (**3**)

To a solution of 0.471 g (2.03 mmol, 1 equiv) of 2 in 10 mL of DCM, the following were added: 0.443 g (2.03 mmol, 1 equiv) of di-tert-butyl dicarbonate and 0.82 mL (6.090 mmol, 3 equiv) of triethylamine. The resulting mixture was stirred at room temperature overnight, and then the volatile compounds were evaporated in a rotary evaporator. The crude product was purified by flash chromatography on silica gel (cyclohexane/EtOAc 6:4 (*v*/*v*)) to give 0.479 g of **3** as a white solid (71%).

Rf 0.40 (cyclohexane/EtOAc 6:4 (*v*/*v*)). ^1^H NMR (300 MHz, CDCl_3_) δ 7.35–7.20 (m, 5H, H_6–10_), 6.68 (br s, 1H, NH), 5.02 (s, 1H, H_3_), 4.34 (t, *J* = 6.7 Hz, 1H, H_2_), 3.17 (dd, *J* = 13.8, 6.7 Hz, 1H, H_4_), 3.03 (dd, *J* = 13.8, 6.7 Hz, 1H, H_4_), 1.30 (s, 9H, H_17–19_), 0.98 (s, 9H, H_12–14_). ^13^C NMR (75 MHz, CDCl_3_) δ 173.7 (C_1_), 156.1 (C_15_), 138.1 (C_5_), 129.7 (C_6_, C_10_), 128.3 (C_7_, C_9_), 126.5 (C_8_), 81.3 (C_16_), 77.5 (C_3_), 61.6 (C_2_), 39.6 (C_4_), 36.7 (C_3_), 28.0 (C_17–19_), 25.8 (C_12–14_). IR (KBr): 3294, 2975, 1721, 1709, 1371, 1357, 1304, 1167, 1076, 953, 805, 780, 749, 737, 702 cm^−1^. HRMS (DCI-CH_4_) Calculated for C_19_H_29_N_2_O_3_ 333.2178 [M+H]^+^, found 333.2174.

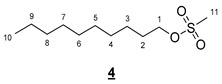

Decyl methanesulfonate (**4**)

To a solution of 5.000 g (31.59 mmol, 1 equiv) of decanol in 50 mL of DCM, the following were added: 3.67 mL (47.38 mmol, 1.5 equiv) of mesyl chloride and 8.53 mL (63.18 mmol, 2 equiv) of trimethylamine. The resulting mixture was stirred for 7 h at room temperature, and then 30 mL of HCl (2N) was added. The medium was vigorously stirred overnight. The organic phase was then washed 3 times with 30 mL of water. The organic phases were combined, dried (MgSO_4_) and concentrated in vacuo to give 7.119 g of **4** as yellow oil (95%). The structure of **4** was confirmed by comparison with literature reported data [31].

^1^H NMR (300 MHz, CDCl_3_) δ 4.22 (t, *J* = 6.6 Hz, 2H, H_1_), 3.00 (s, 3H, H_11_), 1.86–1.65 (m, 2H, H_2_), 1.46–1.16 (m, 14H, H_3–9_), 0.88 (t, *J* = 6.6 Hz, 3H, H_10_). IR (KBr): 2929, 2856, 1467, 1354, 1177, 975, 836, 746, 722 cm^−1^.

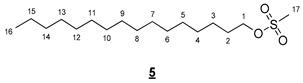

Hexadecyl methanesulfonate (**5**)

To a solution of 4.740 g (19.550 mmol, 1 equiv) of hexadecanol in 30 mL of DCM, the following were added: 2.27 mL (29.330 mmol, 1.5 equiv) of mesyl chloride and 3.16 mL (39.100 mmol, 2 equiv) of pyridine. The resulting mixture was stirred for 7 h at room temperature, and then 20 mL of HCl (2N) was added. The medium was vigorously stirred overnight. The organic phase was then washed with 20 mL of an aqueous solution of NaHCO_3_ (5%) and 20 mL of water. The organic phases were combined, dried (MgSO_4_), and concentrated in vacuo to give 4.380 g of **5** as a white solid (70%). The structure of **5** was confirmed by comparison with literature reported data [31].

^1^H NMR (300 MHz, CDCl_3_) δ 4.22 (t, *J* = 6.6 Hz, 2H, H_1_), 3.00 (s, 3H, H_17_), 1.86–1.64 (m, 2H, H_2_), 1.46–1.16 (m, 26H, H_3–15_), 0.88 (t, *J* = 6.7 Hz, 3H, H_16_). IR (KBr): 2918, 2851, 1474, 1344, 1328, 1168, 1160, 984, 942, 851, 751, 718 cm^−1^.

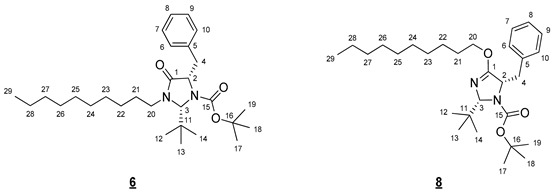

*tert*-Butyl (2*R*,5*S*)-5-benzyl-2-(*tert*-butyl)-4-(decyloxy)-2,5-dihydro-1*H*-imidazole-1-carboxylate (**6**) et *tert*-butyl (2*R*,5*S*)-5-benzyl-2-(tert-butyl)-3-decyl-4-oxoimidazolidine-1-carboxylate (**8**)

To a solution of 1.000 g (3.01 mmol, 1 equiv) of **3** in 20 mL of DMF, 0.241 g (6.02 mmol, 2 equiv) of sodium hydride (60% in mineral oil) was added. The resulting mixture was stirred for 5 min. A solution of 0.712 g (3.01 mmol, 1 equiv) of **4** in 5 mL of DMF was then added and the medium was stirred at 60 °C for 6 h. It was cooled to room temperature and hydrolyzed with 20 mL of water. The resulting aqueous phase was extracted with 50 mL of ethyl acetate. The combined organic phases were washed five times with 20 mL of water, dried (MgSO_4_), and concentrated in vacuo. The crude product was purified by flash chromatography on silica gel (cyclohexane/AcOEt 95:5 (*v*/*v*)) to give 0.404 g (28%) of **6** and 0.581 g (41%) of **8** as colorless oils.

**Compound 6** (Rf 0.19 (cyclohexane/AcOEt 9:1 (*v*/*v*))): ^1^H NMR (300 MHz, CDCl_3_) δ 7.36–7.17 (m, 5H, H_6–10_), 5.12 (s, 1H, H_3_), 4.32 (dd, *J* = 7.2, 6.2 Hz, 1H, H_2_), 3.92 (ddd, *J* = 14.1, 9.3, 7.2 Hz, 1H, H_20_), 3.20 (dd, *J* = 14.1, 6.2 Hz, 1H, H_4_), 3.03 (ddd, *J* = 9.3, 7.2, 3.4 Hz, 1H, H_20_), 2.98 (dd, *J* = 13.8, 7.2 Hz, 1H, H_4_), 1.35–1.22 (m, 23H, H_22–28_, H_17–19_), 1.06 (s, 9H, H_12–14_), 0.87 (t, *J* = 6.7 Hz, 3H, H_29_). ^13^C NMR (75 MHz, CDCl_3_) δ 171.5 (C_1_), 156.0 (C_15_), 138.3 (C_5_), 129.7 (C_6_, C_10_), 128.4 (C_7_, C_9_), 126.5 (C_8_), 81.1 (C_16_), 79.2 (C_3_), 61.9 (C_2_), 42.8 (C_20_), 39.8 (C_4_), 37.4 (C_11_), 31.9 (CH_2_), 29.5 (CH_2_), 29.5 (CH_2_), 29.3 (CH_2_), 29.2 (CH_2_), 28.0 (C_17–19_), 27.0 (C_12–14_), 26.7 (CH_2_), 26.5 (C_21_), 22.7 (CH_2_), 14.1 (C_29_). IR (KBr) 2927, 2856, 1708, 1366, 1167, 786, 751, 699 cm^−1^. HRMS (DCI-CH_4_) Calculated for C_29_H_49_N_2_O_3_ 473.3743 [M+H]^+^, found 473.3761.
**Compound 8** (Rf 0.35 (cyclohexane/AcOEt 9:1 (*v*/*v*))): ^1^H NMR (300 MHz, CDCl_3_) δ 7.33–7.14 (m, 5H, H_6–10_), 5.19 (s, 1H, H_3_), 4.52 (dd, *J* = 7.8, 5.7 Hz, 1H, H_2_), 4.24 (dt, *J* = 10.5, 6.5 Hz, 1H, H_20_), 4.03 (dt, *J* = 10.5, 6.6 Hz, 1H, H_20_), 3.07 (dd, *J* = 13.6, 5.7 Hz, 1H, H_4_), 2.93 (dd, *J* = 13.6, 7.8 Hz, 1H, H_4_), 1.68–1.54 (m, 2H, H_21_), 1.36 (s, 9H, H_17–19_), 1.32–1.22 (m, 14H, H_22–28_), 0.98 (s, 9H, H_12–14_), 0.88 (t, *J* = 6.7 Hz, 3H, H_29_). ^13^C NMR (75 MHz, CDCl_3_) δ 167.6 (C_1_), 156.1 (C_15_), 138.5 (C_5_), 129.6 (C_6_, C_10_), 128.2 (C_7_, C_9_), 126.3 (C_8_), 90.1 (C_3_), 80.3 (C_16_), 68.3 (C_20_), 63.9 (C_2_), 40.2 (C_4_), 36.6 (C_11_), 31.9 (CH_2_), 29.6 (CH_2_), 29.5 (CH_2_), 29.3 (CH_2_), 29.3 (CH_2_), 28.7 (C_21_), 28.3 (C_17–19_), 26.7 (C_12–14_), 25.9 (CH_2_), 22.7 (CH_2_), 14.1 (C_29_). IR (KBr) 3416, 2927, 2856, 1712, 1667, 1366, 1331, 1163, 786, 745, 699 cm^−1^. HRMS (DCI-CH_4_) Calculated for C_29_H_49_N_2_O_3_ 473.3743 [M+H]^+^, found 473.3751.

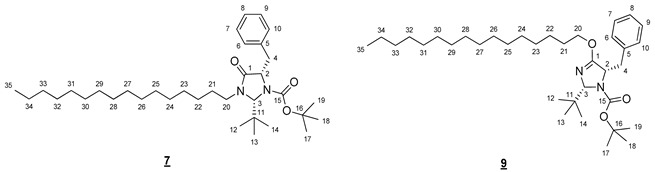

*tert*-Butyl (2*R*,5*S*)-5-benzyl-2-(*tert*-butyl)-4-(hexadecyloxy)-2,5-dihydro-1*H*-imidazole-1-carboxylate (**7**) and *tert*-butyl (2*R*,5*S*)-5-benzyl-2-(tert-butyl)-3-hexadecyl-4-oxoimidazolidine-1-carboxylate (**9**)

To a solution of 1.000 g (3.02 mmol, 1 equiv) of **3** in 20 mL of DMF, 0.242 g (6.04 mmol, 2 equiv) of sodium hydride (60% in mineral oil) was added. The mixture obtained was stirred for 5 min. A solution of 0.967 g (3.02 mmol, 1 equiv) of **5** in 5 mL of DMF was then added and the medium was stirred at 60 °C for 6 h. It was cooled to room temperature and hydrolyzed with 20 mL of water. The resulting aqueous phase was extracted with 50 mL of ethyl acetate. The combined organic phases were washed five times with 20 mL of water, dried (MgSO_4_), and concentrated in vacuo. The crude product was purified by flash chromatography on silica gel (cyclohexane/AcOEt 95:5 (*v*/*v*)) to give 0.497 g (30%) of **7** and 0.596 g (35%) of **9** as colorless oils.

**Compound 7** (Rf 0.17 (cyclohexane/AcOEt 9:1 (*v*/*v*))): ^1^H NMR (300 MHz, CDCl_3_) δ 7.36–7.14 (m, 5H, H_6–10_), 5.12 (s, 1H, H_3_), 4.39–4.27 (m, 1H, H_2_), 3.92 (ddd, *J* = 14.2, 9.3, 7.3 Hz, 1H, H_20_), 3.20 (dd, *J* = 13.8, 6.0 Hz, 1H, H_4_), 3.03 (ddd, *J* = 9.3, 6.7, 3.4 Hz, 1H, H_20_), 2.98 (dd, *J* = 13.8, 7.2 Hz, 1H, H_4_), 1.67 (m, 2H, H_21_), 1.38–1.15 (m, 26H, H_22–34_), 1.26 (s, 9H, H_17–19_) 1.06 (s, 9H, H_12–14_), 0.88 (t, *J* = 6.7 Hz, 3H, H_35_). ^13^C NMR (75 MHz, CDCl_3_) δ 171.5 (C_1_), 156.0 (C_15_), 138.3 (C_5_), 129.7 (C_6_, C_10_), 128.3 (C_7_, C_9_), 126.5 (C_8_), 81.1 (C_16_), 79.2 (C_3_), 61.9 (C_2_), 42.7 (C_20_), 39.8 (C_4_), 37.4 (C_11_), 31.9 (CH_2_), 29.7 (3 * CH_2_), 29.7 (2 * CH_2_), 29.6 (CH_2_), 29.5 (2 * CH_2_), 29.4 (CH_2_), 29.2 (CH_2_), 28,0 (C_17–19_), 27.0 (C_12–14_), 26.7 (C_22_), 26.5 (C_21_), 22.7 (CH_2_), 14.1 (C_35_). IR (KBr) 2926, 2854, 1708, 1367, 1167, 786, 750, 698 cm^−1^. HRMS (DCI-CH_4_) Calculated for C_35_H_61_N_2_O_3_ 557.4682 [M+H]^+^, found 557.4688.
**Compound 9** (Rf 0.38 (cyclohexane/AcOEt 9:1 (*v*/*v*))): ^1^H NMR (300 MHz, CDCl_3_) δ 7.35–7.13 (m, 5H, H_6–10_), 5.19 (s, 1H, H_3_), 4.53 (dd, *J* = 7.7, 5.8 Hz, 1H, H_2_), 4.24 (dt, *J* = 10.5, 6.5 Hz, 1H, H_20_), 4.03 (dt, *J* = 10.5, 6.6 Hz, 1H, H_20_), 3.07 (dd, *J* = 13.6, 5.6 Hz, 1H, H_4_), 2.93 (dd, *J* = 13.6, 7.9 Hz, 1H, H_4_), 1.68–1.55 (m, 2H, H_21_), 1.36 (s, 9H, H_17–19_), 1.32–1.20 (m, 26H, H_22–34_), 0.98 (s, 9H, H_12–14_), 0.88 (t, *J* = 6.7 Hz, 3H, H_35_). ^13^C NMR (75 MHz, CDCl_3_) δ 167.6 (C_1_), 156.1 (C_15_), 138.5 (C_5_), 129.6 (C_6_, C_10_), 128.1 (C_7_, C_9_), 126.3 (C_8_), 90.1 (C_3_), 80.2 (C_16_), 68.3 (C_20_), 63.9 (C_2_), 40.2 (C_4_), 36.6 (C_11_), 32.0 (C_33_), 29.7 (4 * CH_2_), 29.7 (2 * CH_2_), 29.6 (CH_2_), 29.6 (CH_2_), 29.4 (CH_2_), 29.3 (CH_2_), 28.7 (C_21_), 28.3 (C_17–19_), 26.7 (C_12–14_), 25.9 (C_22_), 22.7 (CH_2_), 14.1 (C_35_). IR (KBr) 2925, 2855, 1712, 1666, 1365, 1331, 1175, 786, 745, 698 cm^−1^. HRMS (DCI-CH_4_) Calculated for C_35_H_61_N_2_O_3_ 557.4682 [M+H]^+^, found 557.4700.

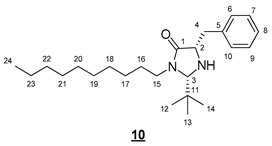

(2*S*,5*S*)-5-benzyl-2-(*tert*-butyl)-3-decylimidazolidin-4-one (**10**)

To a solution of 0.403 g (0.85 mmol, 1 equiv) of **6** in 2.5 mL of DCM, 1.378 mL (17.09 mmol, 20 equiv) of trifluoroacetic acid was added. The resulting mixture was stirred at room temperature for 3 h. The volatile compounds were evaporated in a rotary evaporator. The medium was resolubilized in 3 mL of DCM and then treated with 1 mL of aqueous sodium hydroxide solution (1M). The organic phase was dried (MgSO_4_) and concentrated in vacuo. The crude product was purified by flash chromatography on silica gel (cyclohexane/EtOAc 7:3 (*v*/*v*)) to give 0.262 g of **10** (80%) as a yellow oil.

Rf 0.33 (cyclohexane/AcOEt 7:3 (*v*/*v*)). ^1^H NMR (500 MHz, CDCl_3_) δ 7.31–7.19 (m, 5H, H_6–10_), 4.23 (s, 1H, H_3_), 3.77 (ddd, *J* = 14.0, 9.7, 7.0 Hz, 1H, H_15_), 3.72–3.65 (m, 1H, H_2_), 3.15 (dd, *J* = 13.7, 3.9 Hz, 1H, H_4_), 3.05 (ddd, *J* = 14.0, 9.4, 4.6 Hz, 1H, H_15_), 2.91 (dd, *J* = 13.7, 7.7 Hz, 1H, H_4_), 1.63–1.54 (m, 1H, H_16_), 1.52–1.44 (m, 1H, H_16_), 1.34–1.20 (m, 14H, H_17–23_), 0.88 (t, *J* = 7.0 Hz, 3H, H_24_), 0.84 (s, 9H, H_12–14_). ^13^C NMR (126 MHz, CDCl_3_) δ 175.3 (C_1_), 138.0 (C_5_), 129.6 (C_6_, C_10_), 128.5 (C_7_, C_9_), 126.6 (C_8_), 79.6 (C_3_), 59.3 (C_2_), 42.6 (C_15_), 38.2 (C_4_), 35.4 (C_11_), 31.9 (CH_2_), 29.6 (CH_2_), 29.5 (CH_2_), 29.3 (2 * CH_2_), 27.0 (CH_2_), 26.9 (C_16_), 25.5 (C_12–14_), 22.7 (CH_2_), 14.1 (C_24_). IR (KBr) 2927, 2855, 1698, 1455, 751, 721, 700 cm^−1^. HRMS (DCI-CH_4_) Calculated for C_24_H_41_N_2_O 373.3219 [M+H]^+^, found 373.3213.

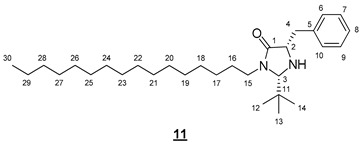

(2*S*,5*S*)-5-benzyl-2-(*tert*-butyl)-3-hexadecylimidazolidin-4-one (**11**)

To a solution of 0.497 g (0.89 mmol, 1 equiv) of **7** in 2.5 mL of DCM, 1.378 mL (17.85 mmol, 20 equiv) of trifluoroacetic acid was added. The resulting mixture was stirred at room temperature for 3 h. The volatile compounds were evaporated in a rotary evaporator. The medium was resolubilized in 3 mL of DCM and then treated with 1 mL of aqueous sodium hydroxide solution (1M). The organic phase was dried (MgSO_4_) and concentrated in vacuo. The crude product was purified by flash chromatography on silica gel (cyclohexane/EtOAc 7:3 (*v*/*v*)) to give 0.270 g of **11** (66%) as a yellow oil.

Rf 0.37 (cyclohexane/AcOEt 7:3 (*v*/*v*)). ^1^H NMR (500 MHz, CDCl_3_) δ 7.31–7.19 (m, 5H, H_6–10_), 4.23 (s, 1H, H_3_), 3.77 (ddd, *J* = 14.1, 9.7, 7.1 Hz, 1H, H_15_), 3.72–3.65 (m, 1H, H_2_), 3.15 (dd, *J* = 13.7, 3.9 Hz, 1H, H_4_), 3.05 (ddd, *J* = 14.1, 9.3, 4.5 Hz, 1H, H_15_), 2.91 (dd, *J* = 13.7, 7.7 Hz, 1H, H_4_), 1.63–1.54 (m, 1H, H_16_), 1.51–1.44 (m, 1H, H_16_), 1.34–1.19 (s, 26H, H_17–29_), 0.88 (t, *J* = 6.9 Hz, 3H, H_30_), 0.84 (s, 9H, H_12–14_). ^13^C NMR (126 MHz, CDCl_3_) δ 175.3 (C_1_), 138.1 (C_5_), 129.6 (C_6_, C_10_), 128.5 (C_7_, C_9_), 126.6 (C_8_), 79.6 (C_3_), 59.3 (C_2_), 42.6 (C_15_), 38.3 (C_4_), 35.4 (C_11_), 31.9 (CH_2_), 29.7 (3 * CH_2_), 29.7 (CH_2_), 29.7 (CH_2_), 29.7 (CH_2_), 29.6 (CH_2_), 29.6 (CH_2_), 29.4 (CH_2_), 29.3 (CH_2_), 27.0 (CH_2_), 26.9 (C_16_), 25.5 (C_12–14_), 22.7 (CH_2_), 14.1 (C_30_). IR (KBr) 2926, 2854, 1699, 1455, 699 cm^−1^. HRMS (DCI-CH_4_) Calculated for C_30_H_53_N_2_O 457.4158 [M+H]^+^, found 457.4177.

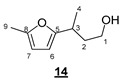

3-(5-Methylfuran-2-yl)butan-1-ol (**14**)

To a solution of 0.027 g (0.07 mmol, 0.1 equiv) of **10** in 2.5 mL of DCM, the following were added: 5.5 µL (0.07 mmol, 0.1 equiv) of trifluoroacetic acid, 329 µL (3.57 mmol, 5 equiv) of 2-methylfuran, and 58 µL (0.71 mmol, 1 equiv) of crotonaldehyde. The resulting mixture was stirred at room temperature for 6 h and then transferred into a vial containing 0.135 g (3.57 mmol, 5 equiv) of NaBH_4_ in 2.5 mL of EtOH. After 15 min of stirring at room temperature, 10 mL of a saturated aqueous NaHCO_3_ solution was added. The resulting medium was extracted three times with 10 mL of DCM. The combined organic phases were then washed with 10 mL of saturated aqueous NaHCO_3_ solution and 10 mL of brine. The organic phase was dried (MgSO_4_) and concentrated in vacuo. The crude product was purified by flash chromatography on silica gel (cyclohexane/EtOAc 9:1 (*v*/*v*)) to give 0.104 g of **14** (95%) as a yellow oil. The structure of **14** was confirmed by comparison with literature reported data.

Rf 0.37 (cyclohexane/AcOEt 7:3 (*v*/*v*)). ^1^H NMR (500 MHz, CDCl_3_) δ 5.86 (d, *J* = 3.0 Hz, 1H, H_6_), 5.84 (d, *J* = 3.0 Hz, 1H, H_7_), 3.71–3.61 (m, 2H, H_1_), 2.94 (m, 1H, H_3_), 2.25 (s, 3H, H_9_), 1.89 (ddt, *J* = 14.0, 8.0, 6.2 Hz, 1H, H_2_), 1.77 (td, *J* = 14.0, 6.6 Hz, 1H, H_2_), 1.25 (d, *J* = 7.0 Hz, 3H, H_4_). ^13^C NMR (126 MHz, CDCl_3_) δ 158.1 (C_5_), 150.3 (C_8_), 105.6 (C_6_), 104.3 (C_7_), 61.0 (C_1_), 38.8 (C_2_), 29.9 (C_3_), 19.4 (C_4_), 13.5 (C_9_). HRMS (DCI-CH_4_) Calculated for C_9_H_15_O_2_ 155.1072 [M+H]^+^, found 155.1073.

### 3.3. Surface Tension Analysis

The surface tension of the surfactants was determined at 25 °C with a GBX instrument using a platinum plate. A 1 g·L^−1^ solution of each surfactant was prepared in ultrapure water. The surface tension of this solution was measured. The solution was then diluted with ultrapure water. Each experimental sample was measured in triplicate and averaged. The relationship curves between surface tension (γ) and logarithmic concentration (Log C) were drawn. In addition, the surface tension of ultrapure water was measured to be 72.00 mN·m^−1^.

### 3.4. Dynamic Light Scattering Tests

The hydrodynamic diameter of surfactant aggregates in aqueous solution (3 g·L^−1^ and 1 g·L^−1^ for molecules **12** and **13**, respectively**)** was determined with a Zetasizer Nano-ZS nanoparticle size potentiometer. The scattering angle is 173° using a helium–neon laser with a wavelength of 633 nm. The samples were kept at 25 °C for more than 5 h prior to the experiment. The aqueous solutions of the surfactants were filtered with a 0.45 mm filter head to remove any impurities. Each analysis was performed in triplicate to ensure the reproducibility of the measurement.

## 4. Conclusions

In conclusion, we have shown in this work that it is possible to synthesize new organocatalytic surfactants. These were obtained from as many biobased compounds as possible, in accordance with the 12 principles of Green Chemistry. The properties of these molecules were studied in water: we were able to demonstrate that they present critical aggregation concentrations, surface tensions at the CAC_w_, and surface adsorption parameters close to those of conventional surfactants. DLS analyses also confirmed that these molecules self-assemble to form vesicles in water. Finally, we tested the asymmetric catalytic activity of these organocatalytic surfactants in Diels–Alder reactions carried out in dichloromethane. The results were encouraging, as the desired molecules were obtained in good yields and enantioselectivities. However, the enantioselectivity decreased when the reaction was carried out in water. Work is currently underway to improve these results, as this methodology would eventually allow the synthesis of asymmetric molecules with high added value in water, which is a current challenge for many chemical sectors.

## Figures and Tables

**Table 1:**
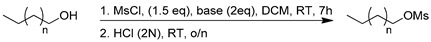
Synthesis of mesylated alcohol **4** and **5**.

Fatty Alcohol	Base	Compound	Yield (%)
Decan-1-ol (n = 7)	Triethylamine	**4**	95
Hexandecan-1-ol (n = 13)	Pyridine	**5**	70

**Table 2:** Critical association concentration (CAC_w_), surface tension at the CAC (γ_w_), and surface adsorption parameters (Γ_max_, A_min_) for compounds **12 **and **13 **and three other conventional surfactants.

Surfactant	12	13	Tween 20	Triton X 100	CTAB
CAC_w_ (g·L^−1^)	0.12	0.25	0.10 ^b^	0.27 ^c^	0.33 ^c^
γ_w_ (mN·m^−1^)	49	39	41 ^b^	30 ^a^	34 ^e^–35 ^c^
Γ_max_ (µmol·m^−2^)	2.32	2.28	2.8 ^d^	2.1 ^c^	2.4 ^f^–2.6 ^c^
A_min_ (A^2^·molecule^−1^)	71.6	72.8	58 ^b^–59.5 ^d^	68 ^c^	63 ^c^–69.2 ^f^

^a^ Reference [24]; ^b^ Reference [25]; ^c^ Reference [26]; ^d^ Reference [27]; ^e^ Reference [28]; ^f^ Reference [29].

## Data Availability

Data are contained within the article.
